# Identification and tissue expression profiling of odorant binding protein genes in the red palm weevil, *Rhynchophorus ferrugineus*

**DOI:** 10.1186/s40064-016-3198-x

**Published:** 2016-09-13

**Authors:** Wei Yan, Li Liu, Weiquan Qin, Youqing Luo, Xuezhong Ma, Nabil Haider, Muhanad Inayeh

**Affiliations:** 1 Key Laboratory for Silviculture and Conservation of Ministry of Education, Beijing Forestry University, Beijing, 100083 China; 2 Coconut Research Institute, Chinese Academy of Tropical Agricultural Sciences, Hainan, 571339 China; 3 China-Arab Date Palm Research Center, Ningxia, 750001 China

**Keywords:** Expression pattern, Odorant binding protein, Olfactory, *Rhynchophorus ferrugineus*

## Abstract

**Background:**

The red palm weevil, *Rhynchophorus ferrugineus,* is a lethal pest of the palms. The identification of odorant binding protein (OBP) genes will be helpful for clarifing the mechanism of odorant detection of this pest. By sequencing the full length cDNA library of its antenne, 11 OBP genes (RferOBP1-11) were identified.

**Findings:**

The result showed RferOBP1-7 and RferOBP8-11 belonged to the minus-C and classic family, respectively qPCR revealed that RferOBP1-10 highly transcribed in the antennae, of which RferOBP1, RferOBP4, RferOBP8 and RferOBP10 were obviously male-biased expression. RferOBP7 and RferOBP11 exhibited highly expression in female head and male thorax. RferOBP2, RferOBP5 and RferOBP6 were highly expressed in the female thorax, leg and abdomen respectively.

**Conclusions:**

The results paved the way towards a future understanding of the olfaction in this species.

## Background

The red palm weevil, *Rhynchophorus ferrugineus*, belongs to Coleoptera: Dryophthoridae family and is native to tropical Asian regions which have become a most threatening pest of palm trees worldwide (Yan et al. 2015). *R. ferrugineus* is a concealed tissue borer that resides inside the trunk of the palms with a highly aggregated population distribution pattern and tree injuries are often severe when it is discovered (Abraham et al. 1998). The widespread nature of this pest is due to their ability of adoption to a broader host range, to a wider variety of climates, and practice of shipping the palm trees between different territories (Murphy and Briscoe 1999). In the newly invaded areas, this pest generally causes serious damage to the trees and significant economic losses are associated by minimizing the production of palms, and increases cost management in eradication of the pest (El-Sabea et al. 2009). Due to its concealed nature and exceptional colonization capability, integrated pest management strategies play an important role in controlling the red palm weevil (Vacas et al. 2014). With respect to this, large quantities of insecticides are still applied to prevent and control *R. ferrugineus* infestations (Llácer et al. 2013). However, trapping systems which include aggregation pheromone, date fruits, bait traps have been effectively and environmentally used to detect and control this pest (Abuagla and Al-Deeb 2012). New management strategies from the aspect of chemical ecology should be developed by understanding the *R. ferrugineus* olfactory system, which will be efficient in reducing the weevil populations and the usage of pesticides. However, most of the olfactory genes have been deciphered by transcriptome sequencing are fragmental and their expression patterns are not well known (Yan et al. 2015; Antony et al. 2016). Thus, the information regarding the molecular mechanisms underlying the olfaction in this species is scarce.

Odorant-binding proteins (OBPs) are hydrophilic soluble proteins which are composed of approximately 130 amino acids and typically contain six conserved cysteine residues (Leal et al. 1999) which are secreted by the accessory cells around the olfactory neurons and are accumulated in the sensillar lymph (Klein 1987). OBPs are essential for insect olfaction which acts by transporting hydrophobic compounds through aqueous sensillar lymph to the receptors embedded on dendritic membranes of olfactory receptor neurons from the external environment and is thought to be the first step in the recognition of chemical signals (Krieger and Breer 1999; Leal and Leal 2015). Hence, it is necessary to understand the mechanism of olfaction of *R. ferrugineus* OBPs which has an important role. Hence, in our study we reported the identification of 11 OBP genes from the antennae of this pest and analyzed their tissue expression patterns.

## Methods

### Insects and sample collection

*Rhynchophorus ferrugineus* was collected from cocoons but was originally collected in naturally infested palms in the suburbs of Hainan province. The lab colony was maintained as described in the study by Yan et al. (2014). The antennae, heads (without antenna), thoraxes, abdomens, and legs of male and female were collected separately and frozen immediately in liquid nitrogen.

### cDNA library construction and sequencing

Total RNA was extracted from approximately 100 antennae of both sexes after 5 days of emergence by Trizol regent. The quantity and quality of the total RNA was validated using spectrophotometer. 5 μg of total RNA was subjected to construct full length cDNA library using Creator SMART cDNA Library Construction Kit (Clontech, Mountain View, CA, USA) according to the manufacturer’s instructions. During the library construction, 400–1200 bp fragments were selected. About 1000 clones were randomly sequenced from 5′-end of the gene. After BlastX annotation, the genes were assigned to the OBP family and were completely sequenced to obtain their full length cDNA. The signal peptide of the protein sequences were predicted using SignalP 4.0 server version (Petersen et al. 2011). Sequence alignments were performed using the program ClustalX (Thompson et al. 1997). Phylogenetic trees were constructed by MEGA version 5 based on the neighbor-joining algorithm method at bootstrap 1000 (Tamura et al. 2011).

### qPCR analysis

Total RNA from different tissues were extracted as mentioned above. After DNase treatment, the cDNA was synthesized from 1 μg total RNA using a GoScript Reverse Transcription System (Promega, USA). qPCR was performed using gene-specific primers (Table 1) by SYBR Premix EX Taq™ (TaKaRa, Dalian, Liaoning, China) in three biological and technical replicates with different samples. PCR reactions were performed in a 3-step amplification process at 94 °C for 30 s, 55 °C for 30 s, and 72 °C for 30 s. Expression levels of the genes were calculated relative to the control gene 18S RNA expression using the $2^{{ - \Delta \Delta {\text{C}}_{T} }}$ method (Livak and Schmittgen 2001). The total expression of one gene in all the tissues was set as 100 % and the percentage of one gene in each tissue was used to measure the expression level.

**Table 1:** qPCR primers

Primer name		Sequence (5′ → 3′)
RferOBP1	Reverse	TCCTCGCCCAACATTAC
	Forward	TTTGACCGCCTCCTTTA
RferOBP2	Reverse	TAGTCCAAGCGGATCTCACA
	Forward	CGTAGCACCAGTTTCCTC
RferOBP3	Reverse	TTTTCAGCGACTCACCA
	Forward	GACATTTATCTAGCATAGCG
RferOBP4	Reverse	TGGAGAACTCACCGACTC
	Forward	CGAACAACATAATCCCTTT
RferOBP5	Reverse	CTGAACAACGCCAGAGG
	Forward	TCATTCCCAAACATACCA
RferOBP6	Reverse	CTGAACAACGCCAGAGG
	Forward	TCATTCCCAAACATACCA
RferOBP7	Reverse	TGGTGTCGGCCATCTCA
	Forward	CTTCGCCCTCGTCGTTT
RferOBP8	Reverse	TGATGGTATGTGGGACTT
	Forward	ATGGTGGAGCCTGAGTT
RferOBP9	Reverse	AGGCGACTGGGAGGTAG
	Forward	TGTGCGTCTGCGGATTT
RferOBP10	Reverse	GGTACTCCTCGCTGTTT
	Forward	ATCCATAGATCCCGTTT
RferOBP11	Reverse	AACAGGAGCAACAGAAGAT
	Forward	ATTACTGGCGGTAGGGT

## Results and discussion

### Characteristics of OBP sequences

**Fig. 1:**
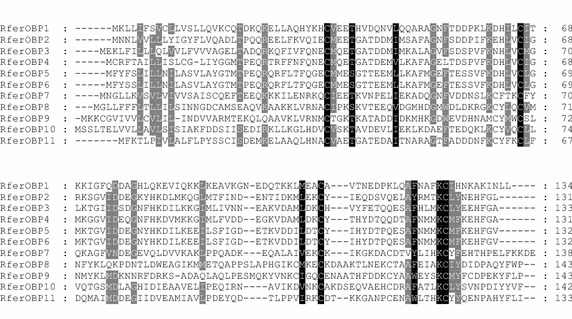
Alignment of the amino acid sequences of *Rhynchophorus ferrugineus* OBPs

**Fig. 2:**
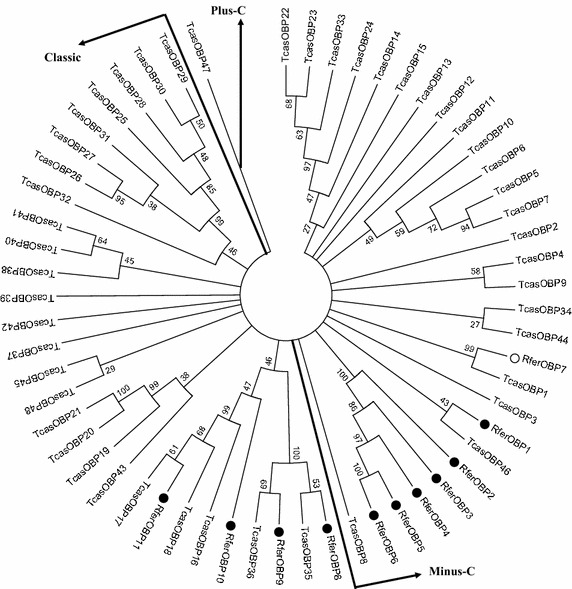
Neighbor-joining tree of OBP proteins from *Rhynchophorus ferrugineus* and *Tribolium castaneum*. The phylogenetic tree was presented with 25 % cut-off bootstrap value

In total, 11 full length OBP genes from 36 clones named as RferOBP1-11 were identified from the antennal cDNA library sequencing, which have been deposited in the NCBI under the accession numbers KR780571 to KR780581 (Table 2). Compared to the 49 OBPs identified in the genome of *Tribolium castaneum*, more OBP genes might be expected to be identified in *R. ferrugineus* by massive sequencing strategies and by using more different tissues that may also express OBPs (Tribolium Genome Sequencing Consortium 2008; Zhu et al. 2013). The cDNA length ranged from 497 bp to 579 bp. The open reading frame (ORF) ranged from 396 to 441 bp, which encoded approximately 130 amino acid residues, with a predicted molecular weight of about 15 kDa. Except for RferOBP1, 8 and 9, the predicted pH of others were acidic. The amino acid sequences identity of RferOBP5 and RferOBP6 was 99 %, while their DNA sequence homology was only 62 % (Fig. 1). RferOBP5 and RferOBP6 might be the result of gene duplication events. Similar to the characteristics of OBP with a rather low level of sequence similarity (Forêt and Maleszka 2006; Zhu et al. 2012), RferOBP1-4 and RferOBP7-11 shared 8–65 % amino acid residual identities with each other. In addition, they contained the characteristic sequence features for these proteins, such as conserved cysteine residues and signal peptide. All identified OBPs were used to construct a phylogenetic tree clustering with OBPs from *T. castaneum* (Fig. 2), which was the only species with known genomic sequence from Coleoptera and were distributed into all three groups. The phylogenetic tree had relatively large minus-C and classic OBP, and one plus-C OBP branches. Minus-C OBPs lack the second and fifth cysteine residues, and plus-C OBPs contained three additional conserved cysteines and one conserved proline (Xu et al. 2009). Four (RferOBP8-11) of the candidate sequences represented classic OBPs, each containing the six conserved cysteine residues. RferOBP1-7 showed minus-C OBPs, and there was no plus-C OBP. Of the 12 OBPs, RferOBP2-6 were clustered with each other together, rather than those from other insect species suggesting that they may be the products of recent gene duplications (Li et al. 2015a, b). These OBPs from different clades may have different functions, which are interesting to be determined.

**Table 2:** Characteristics of *Rhynchophorus ferrugineus* OBP genes

Gene name	Nucleotide length (bp)	ORF length (bp)	Complete protein (aa)	Signal peptide (aa)	Molecular weight (kDa)	Isoelectric point	Accession number
RferOBP1	556	405	135	19	15.27	8.43	KR780571
RferOBP2	569	396	132	18	15.18	4.42	KR780572
RferOBP3	568	402	134	20	15.03	5.25	KR780573
RferOBP4	497	396	132	18	14.86	5.02	KR780574
RferOBP5	509	399	133	17	15.21	4.43	KR780575
RferOBP6	533	399	133	17	15.16	4.43	KR780576
RferOBP7	580	441	147	19	16.68	6.59	KR780577
RferOBP8	556	432	144	18	16.16	7.01	KR780578
RferOBP9	579	432	144	21	15.56	7.78	KR780579
RferOBP10	570	429	143	19	15.82	4.6	KR780580
RferOBP11	571	402	134	18	14.96	4.29	KR780581

### Tissue expression pattern

To uncover the tissue expression pattern, we used qPCR to assess the transcriptional abundance of the identified 11 OBP genes which showed varying degrees of expression in the antennae, head (without antenna), thorax, abdomen, and leg (Table 3). Except for RferOBP11 with abundant expression in female head and male thorax, RferOBP1-10 showed a relatively higher expression in the antennae suggesting that the OBP genes identified in current study may play an important role in the olfaction (Leal 2013). Of them, RferOBP1, RferOBP4, RferOBP8 and RferOBP10 were obviously male biased which may play the same role as pheromone binding proteins and could play a role in odorant perception of certain plant volatiles (Zhang et al. 2015). RferOBP3 was highly expressed in the legs with near identical expression with the antennae. With respect to the other genes, RferOBP2, RferOBP5 and RferOBP6 were highly expressed in the female thorax, leg and abdomen, respectively. As head, thorax and leg have taste sensilla and other olfactory sensilla, OBP expressed in these tissues may function in the perception of non-volatile host chemicals, gustatory reorganization and other olfactory function (Mitaka et al. 2011). These results were similar to the previous studies which showed that OBPs of some insect are expressed primarily or exclusively in non-antennae tissues or in larvae, which may have physiological functions independent of olfaction (Li et al. 2015a, b). This also reflects that the identification of these OBPs solely depend on the structural similarities and not function. Nonetheless, why OBPs are expressed in abdomen remains a mystery. RferOBP7 exhibited similar expression patterns with that of RferOBP11 and was expressed at a very high level in the female head and male thorax. The expression patterns make an important contribution to our understanding of OBPs in *R. ferrugineus* and might facilitate for their future functional characterization.

**Table 3:** Tissue expression pattern of *Rhynchophorus ferrugineus* OBP genes

Gene	Female					Male				
	Anntena	Head	Thorax	Abdomen	Leg	Anntena	Head	Thorax	Abdomen	Leg
RferOBP1	33.21 ± 1.01	2.66 ± 0.22	0.45 ± 0.03	0.24 ± 0.04	0.16 ± 0.01	61.54 ± 1.23	0.07 ± 0.01	1.12 ± 0.02	0.39 ± 0.04	0.17 ± 0.01
RferOBP2	18.06 ± 1.54	9.88 ± 0.26	23.67 ± 0.01	0.93 ± 0.00	17.17 ± 0.01	11.04 ± 1.68	0.71 ± 0.03	4.24 ± 0.12	1.01 ± 0.01	13.28 ± 0.02
RferOBP3	29.79 ± 0.07	5.83 ± 0.44	0.20 ± 1.11	1.05 ± 0.16	4.96 ± 0.79	29.53 ± 0.75	1.74 ± 0.04	0.02 ± 0.22	0.33 ± 0.12	26.55 ± 0.40
RferOBP4	24.91 ± 1.15	5.11 ± 0.82	4.07 ± 0.01	0.50 ± 0.13	10.45 ± 0.22	39.00 ± 2.14	1.11 ± 0.09	0.90 ± 0.00	0.92 ± 0.03	13.04 ± 0.58
RferOBP5	15.05 ± 0.54	1.07 ± 0.21	5.65 ± 0.29	15.94 ± 0.05	35.88 ± 0.47	12.86 ± 1.28	0.40 ± 0.17	1.06 ± 0.02	0.47 ± 0.13	11.62 ± 1.28
RferOBP6	15.30 ± 1.94	1.47 ± 0.08	1.03 ± 0.29	27.38 ± 1.87	15.27 ± 2.67	22.20 ± 1.10	0.04 ± 0.04	2.99 ± 0.04	2.48 ± 0.04	11.84 ± 1.09
RferOBP7	12.30 ± 1.33	28.06 ± 0.08	2.38 ± 0.07	0.91 ± 4.53	1.78 ± 0.32	9.05 ± 0.98	1.31 ± 0.01	28.85 ± 0.35	8.87 ± 0.16	6.50 ± 1.79
RferOBP8	30.21 ± 0.71	2.18 ± 1.18	0.39 ± 0.16	0.16 ± 0.12	0.09 ± 0.16	65.51 ± 1.23	0.05 ± 0.18	0.83 ± 2.40	0.45 ± 0.50	0.13 ± 0.93
RferOBP9	30.92 ± 1.37	17.68 ± 0.13	0.78 ± 0.04	3.20 ± 0.02	4.73 ± 0.01	23.60 ± 1.35	0.83 ± 0.01	5.44 ± 0.04	8.83 ± 0.04	4.00 ± 0.00
RferOBP10	32.84 ± 0.24	10.23 ± 1.17	0.03 ± 0.04	0.58 ± 0.20	3.64 ± 0.66	43.84 ± 1.11	1.51 ± 0.18	0.22 ± 0.86	4.08 ± 0.85	3.03 ± 0.33
RferOBP11	1.17 ± 0.09	48.84 ± 2.22	3.60 ± 0.03	2.92 ± 0.18	0.52 ± 0.02	3.48 ± 0.11	1.99 ± 0.18	36.33 ± 2.14	0.30 ± 0.03	0.85 ± 0.13

## Conclusion

The work presented here brings an identification of 11 OBP genes from red palm weevil by sequencing the antennal full length cDNA library. In particular, the expression profile studies provided a clear map of these genes, which may further facilitate other functional studies on these genes. These data enables a basis to reveal the molecular mechanisms of olfactory functions in the red palm weevil.
